# Understanding perceived value in tourism: Insights from destinations facing crises

**DOI:** 10.1371/journal.pone.0331144

**Published:** 2025-09-02

**Authors:** Laurent Yacoub, Samer ElHajjar, Youssef Zgheib, Nada Jabbour Al Maalouf

**Affiliations:** 1 Faculty of Business Administration, Holy Spirit University of Kaslik, Lebanon; 2 Department of Marketing, National University of Singapore, Singapore; 3 Faculty of Business Administration and Economics, Notre Dame University, Lebanon; 4 Faculty of Business Administration, Holy Spirit University of Kaslik, Lebanon; Guilin University of Technology, CHINA

## Abstract

Tourism is a significant contributor to global economic growth, underscoring the importance of understanding visitors’ perceptions of attractions in destinations that face challenges and crises. This study investigates key factors influencing tourists’ perceived value of a destination, using the context of such challenging economic and political crises. It aims to determine how tourists evaluate their experiences in these destinations and how these perceptions affect their satisfaction and loyalty. By employing a quantitative method using a structured questionnaire distributed to 784 international tourists visiting Lebanon, the research measures perceived value and satisfaction, considering various destination attributes. The findings reveal that the inherent qualities of a destination significantly impact visitor evaluations, with value for money, competitive pricing, and high-quality experiences enhancing the likelihood of repeat visits and recommendations. Additionally, fun and family experiences play a crucial role in shaping perceived value. Contrary to expectations, knowledge and novelty do not significantly influence perceived value. This paper adds to the tourism literature by providing valuable insights into how tourists perceive value in crisis-prone destinations and addressing the resilience of the tourism sector in volatile regions which provides strategies for managers in such destinations. With its innovative approach, this study integrates quantitative analysis, highlighting the key factors that sustain tourists’ satisfaction and loyalty despite external obstacles.

## Introduction

Tourism is a critical sector in economies worldwide, contributing significantly towards development, employment, and even cultural exchange [1]. As global competition among tourist destinations intensifies, understanding the factors influencing visitors’ decisions and behaviors becomes essential [2]. One of these factors is tourists’ perceived value, which influences their overall satisfaction and the extent to which they are loyal to a destination [3]. Before visiting a destination, people typically assess its benefits, considering what they receive in terms of experiences and services in comparison to the costs they incur. This perception allows tourists to take into account both tangible and intangible elements that may affect their visit, including the quality of attractions and accommodation [4].

Perceived value is a multidimensional construct, shaped by factors such as destination attributes, knowledge, novelty, curiosity, value for money, and recreational experiences [5,6,7,8]. Tourists tend to choose destinations where they perceive greater value through unique cultural experiences, high service quality, and family-friendly environments [4]. Conversely, destinations perceived as costly or risky may deter visitors, as they assess not only the monetary cost but also non-monetary aspects such as effort, time, and perceived safety [3]. Additionally, satisfaction is largely dependent on whether a destination meets or exceeds expectations, influencing whether tourists return and recommend it to others [9]. The relationship between perceived value, satisfaction, and loyalty is well established in tourism literature, yet limited research has explored this dynamic in destinations facing crises.

This study addresses this gap by examining the determinants of perceived value in crisis-affected destinations, where tourists’ evaluations may be shaped by distinct considerations such as destination resilience, affordability, and safety concerns [10,11]. The interplay between crisis conditions and tourism behavior remains underexplored, particularly in Middle Eastern countries where political and economic instability poses challenges for the sector [12]. Tourists continue to visit such destinations for various purposes, including religious tourism, cultural exploration, and leisure travel [13,14]. While crises can negatively impact tourism demand, certain segments of travelers remain motivated to visit due to cultural, religious, and economic reasons. Religious tourism is a significant driver of travel to crisis-affected regions, as spiritual and faith-based experiences often take precedence over concerns about political instability [15]. Many destinations in the Middle East and North Africa attract Muslim travelers engaging in Halal tourism, which includes visiting Islamic heritage sites, mosques, and pilgrimage locations [16,17,18,19]. Similarly, Christian pilgrimage tourism in Lebanon and the Holy Land continues despite regional instability, as faith-based travelers prioritize religious fulfillment over perceived risks [20]. However, economic instability and security concerns may alter their perception of value, impacting satisfaction and loyalty [21,2].

Despite these challenges, crisis-affected destinations continue to attract visitors who contribute to local economies through spending on accommodation, dining, shopping, and entertainment. Tourism plays a vital role in employment generation and economic recovery, making it crucial to understand how perceived value can be enhanced and sustained under adverse conditions. Tourists’ risk perceptions influence their willingness to visit unstable regions, particularly when traveling from safer environments [22]. However, little is known about the specific drivers of perceived value that encourage tourists to overlook crisis-related concerns and still choose such destinations. This study aims to examine the key factors influencing tourists’ perceived value in destinations facing crises, with a specific focus on Lebanon, and how their satisfaction and loyalty are affected.

Understanding these dynamics is particularly relevant in today’s tourism landscape, where many regions are experiencing heightened instability due to economic downturns, geopolitical tensions, and safety concerns. Given the increasing instability in several global tourism markets, particularly in the Middle East, understanding how destinations can maintain their attractiveness and retain visitors despite crises is of critical importance for tourism stakeholders and policymakers. This research contributes to the existing literature by offering empirical insights into tourism resilience, identifying key factors that sustain perceived value despite external challenges, and providing practical recommendations for destination managers, policymakers, and tourism stakeholders.

The paper is organized into four key sections. It begins with a literature review that examines current studies on tourist behavior and perceived value. Following this, the research model is detailed, including hypotheses and methodology. The findings and discussion section then presents and interprets the study’s results. Lastly, the implications section provides practical recommendations for tourism stakeholders and suggests areas for future research.

## Literature review

### Understanding destinations in crises and challenges

Destinations facing crises and challenges are distinct from more stable tourism markets in several critical ways. The most prominent difference lies in the heightened uncertainty and risk associated with these destinations. Unlike stable destinations where tourists can generally expect a consistent and safe experience, crisis-affected areas often confront significant unpredictability. This unpredictability can stem from ongoing political instability, economic hardship, social unrest, environmental disasters, or health emergencies, each of which can profoundly influence the destination’s appeal and the tourist experience [23,24].

Tourists visiting these destinations typically prioritize different factors when making travel decisions. While in stable environments, elements like novelty, educational experiences, and luxury may drive tourist choices, in crisis-affected areas, tourists are more likely to focus on safety concerns, value for money, and the availability of reliable, quality experiences [25]. Religious tourism remains one of the most resilient forms of travel, with pilgrimages continuing despite political or economic instability [26,27]. Research on Halal tourism suggests that many Muslim travelers prioritize religious sites, even in crisis-affected destinations, where tailored services align with their spiritual and cultural needs [15]. Furthermore, leisure-seeking tourists may find relaxation opportunities in crisis-affected destinations, where economic instability makes luxury and wellness tourism more affordable [28,29]. While crises may deter some tourists, others continue to visit destinations where perceived stability exists within specific areas [30]. Governments often enhance security in tourist zones, ensuring safe, high-quality experiences for visitors despite broader instability [31]. Moreover, economic crises can increase the affordability of luxury and premium experiences, attracting tourists seeking high value for money [30]. While crisis-affected destinations may appear unsafe, many tourists perceive specific areas as stable and secure, particularly tourist-friendly zones that receive heightened government protection [30,31]. Additionally, economic instability can enhance the perceived value of travel, as tourists can access high-quality experiences at lower costs [30].

Emotional aspects become more critical, as tourists seek a sense of normalcy and comfort amidst the broader instability [32]. Moreover, the marketing and management strategies that might be effective in stable destinations often require significant adaptation in crisis contexts to address the unique challenges and perceptions associated with these areas [33].

Crisis-affected destinations also face the challenge of overcoming negative perceptions and reputational damage. These destinations must work harder to restore and build confidence among potential visitors, often needing to implement robust crisis management and communication strategies [10]. Additionally, the infrastructure in these areas might be compromised, requiring targeted investment to rebuild and improve tourist facilities [34]. The resilience and adaptability of both the destination and its tourism sector are often tested as they navigate the complexities of recovery and growth amidst ongoing challenges [35].

Lebanon exemplifies a destination that has faced multiple, overlapping crises, making it a unique context for studying tourism in challenging environments. The country has a rich cultural heritage, stunning natural landscapes, and a vibrant social scene, all of which are highly attractive to tourists. However, Lebanon has also been significantly impacted by political instability, economic crises, and social unrest, particularly in recent years [36]. Its economy has been severely affected by a protracted financial crisis, marked by the devaluation of its currency, hyperinflation, and widespread poverty. This economic turmoil has transformed the country into a more affordable destination for international tourists, but it has also created challenges in maintaining the quality of services and infrastructure [37,38]. The political landscape in Lebanon is characterized by frequent instability, government paralysis, and regional tensions, all of which contribute to a sense of uncertainty that can deter potential visitors [39].

### Tourists’ perceived value

The significant attention given to consumer value in the existing literature explains the significance of this concept in marketing research [40,41,42,43]. Researchers believe that consumer value includes two dimensions of consumer behavior which are the economic value and the psychological value [44,45,46,47]. Economic value refers to the financial benefits that customers perceive such as cost savings, price competitiveness, and value for money. Psychological value refers to the intangible emotional benefits such as well-being, emotional state, and personal satisfaction [48].

Furthermore, consumer value is inseparably related to consumer behavior concepts such as satisfaction and quality [49,50]. Many marketing researchers, whether researching tourism literature or not, discussed the differences between satisfaction and quality and the majority found that all is related to the value concept [6,51,52].

When discussing customers’ service assessment, the perceived value was found to be the most significant [53,54,55,56,57,58]. The most universally accepted definition of perceived value inside and outside tourism literature is the one given by Zeithaml [59] who defined consumer value as “the overall assessment of the utility of a product based on the perceptions of what is received and what is given”.

Within the context of tourism, many studies such as Chen and Chen [60]., Ryu et al. [61], and Lee et al. [62] discussed that the perceived value of tourists takes several dimensions including the evaluation of the service’s tangibles, reliability, responsiveness, and assurance, cost-benefit analysis, emotional value, social value, and functional value. Tangibles are the physical aspects of the service, and in tourism, examples include the cleanliness and design of destinations. Reliability refers to the ability of the service provider to deliver promised services dependably and accurately such as on-time transportation services, honored reservations, and accurate tour guides. Responsiveness involves the willingness and ability of service providers to help tourists and respond promptly to their needs and inquiries. Assurance is the degree to which tourists feel confident and secure about the service provided such as the professionalism and knowledge of tour guides. Cost-benefit analysis includes the evaluation of the service in terms of its price relative to the benefits received. Emotional value refers to the feelings or affective states that the tourism experience evokes in tourists. Social value refers to the social benefits that tourists gain from the experience, such as increased social status, prestige, or the ability to connect with others. Finally, functional value relates to the practical or utilitarian benefits of the service such as the usefulness, convenience, and efficiency of the tourism experience such as the ease of booking a tour, the functionality of travel apps, or the convenience of transportation options.

Tourism empirical studies compared to other fields focused on perceived value and the relationship between quality, value, satisfaction, and loyalty [5,6,63,4,64,65]. Petrick [66] initiated a value structure of five dimensions which are behavioral price, monetary price, emotional response, quality, and reputation, and other authors such as Gallarza and Saura [67], Ge et al. [68], and Ladhari [69] used the same structure in the services sector. Tourism experiences are often more holistic and immersive than other service sectors, incorporating multiple elements like travel, accommodation, entertainment, and cultural experiences [70]. The interplay between these elements can influence perceived value in ways that are distinct from other service sectors, where consumption might be more straightforward or transactional [71]. For example, emotional responses to cultural immersion or the reputation of a destination could play a more significant role in perceived value in tourism than in other fields.

Despite the growing body of literature on tourism and perceived value, there remains a significant theoretical gap in understanding how tourists assess value in destinations facing challenges and crises. Much of the existing research on perceived value has focused on stable or well-established tourism markets, where factors such as novelty, luxury, and educational experiences often play central roles in shaping tourist perceptions [72]. However, these factors may not be as relevant or influential in crisis-affected destinations, where tourists are likely to prioritize different elements. The current theoretical models do not adequately account for how crises reshape the tourist experience and alter the dimensions of perceived value. This gap calls for a better understanding of perceived value that considers the unique conditions and constraints of destinations in crisis.

### Theoretical framework

Tourists’ decision-making, satisfaction, and loyalty are shaped by several well-established theories in consumer behavior and tourism research. This study applies four key theoretical frameworks to provide a foundation for understanding how tourists assess value in unstable destinations and how their satisfaction and likelihood of returning are impacted.

First, the Perceived Value Theory suggests that consumers evaluate the worth of a product or service based on the trade-off between benefits received and sacrifices made [59]. In the tourism context, perceived value is influenced by factors such as service quality, cost, destination attributes, emotional experience, and overall trip satisfaction [5,60]. The perceived value concept is particularly relevant in crisis-affected destinations, where tourists must weigh potential risks and uncertainties against the benefits of visiting [22]. This theory forms the foundation of the study, as it examines which factors contribute most to tourists’ perceived value in Lebanon, a destination with both strong cultural appeal and ongoing socio-economic challenges.

Second, the Expectancy-Disconfirmation Theory (EDT) explains how consumer satisfaction and loyalty are formed based on the comparison between initial expectations and actual experiences [73]. If a tourist’s experience exceeds expectations, they are satisfied, whereas if the experience falls short, they are dissatisfied. This theory is particularly relevant in crisis tourism, as visitors may adjust their expectations due to media coverage, perceived risks, or negative perceptions of a destination [13]. When their experience is better than expected, satisfaction and loyalty increase despite the challenges present in the destination.

Third, the Push-Pull Theory explains the motivations behind travel choices, distinguishing between push factors (internal motivations) and pull factors (destination-specific attractions) [74]. Push factors might include curiosity, escape, relaxation, and the desire for family experiences, which drive people to travel while pull factors might include a destination’s cultural heritage, affordability, nightlife, and natural beauty, which attract tourists [75]. In the context of crisis-affected destinations, tourists may reassess their motivations, balancing personal desires against concerns over safety and economic conditions [14].

Finally, Iso-Ahola’s Seeking and Escaping Motivational Theory [76] explains that travel behavior is driven by two psychological motivations which are seeking motivation and escaping motivation. Tourists travel to seek novelty, relaxation, cultural experiences, or religious fulfillment [77]. This is particularly relevant in crisis-affected destinations where travelers may be drawn to historical landmarks, spiritual experiences, or budget-friendly tourism opportunities. Further, tourists travel to escape routine, stress, political instability, or economic constraints in their home countries. Some travelers may view a crisis-affected country as a more affordable or even safer alternative than their own unstable environment. This theory is particularly relevant to crisis-affected destinations, as it helps explain why some tourists still visit these places despite perceived risks. For example, Lebanon attracts visitors who seek religious experiences, historical exploration, and luxury tourism at reduced costs, while others may escape economic pressures or political instability in their home countries by choosing affordable travel.

Being founded on those theories, this study provides a comprehensive framework for understanding tourism behavior in crisis contexts, bridging gaps in prior research that primarily focused on stable tourism markets.

### Drivers of tourists’ perceived value

Tourists’ perceived value is influenced by various factors that can shape their satisfaction and experience [78]. Many authors believe that quality of service has a significant impact on tourists’ perceived value [5,60,6,63,4,64,7,8,65]. Good customer service and personalized experiences contribute to a positive perceived value.

#### Knowledge and novelty and their impact on perceived value in tourism.

A destination image is a tourist’s impression and knowledge about a certain destination, including impressions, ideas, and beliefs [79]. Knowledge and destination image help tourists decide whether the destinations match what they want [80]. Cognitive destination image formation, particularly among first-time visitors, is influenced by their exposure to information and perceptions of novelty and cultural richness [81]. Thus, knowledge and destination image impact tourists’ decisions and choices. Novelty is the quality of being new, unique, or infrequent. Novelty helps understand some multifaceted human motivations because it appeals to sensation-seeking [75]. Tourists intend to explore novel and different travel experiences. Tourists seek to pursue new experiences that break their routines, alleviate their boredom, and cause thrill. Those three factors are usually considered to measure tourists’ novelty [75]. Further, cultural elements, such as heritage, traditions, and architecture, help develop tourists’ knowledge and expectations about a destination, which in turn enhances their perceived value [82]. Tourists visiting culture-based attractions often perceive a higher value in destinations that offer unique and immersive experiences [83]. In crisis-affected destinations, where external perceptions might be negative, strong cultural identity and authentic experiences can counterbalance risks and increase perceived value by reinforcing a destination’s uniqueness [14]. Previous studies found that tourists’ knowledge and destination image has an important and positive impact on perceived value and novelty [84,85,86,87,88,6,89]. Informed tourists are more likely to perceive a destination favorably while novelty-seeking travelers value new, unexpected, and engaging experiences that make a destination stand out.

As noted by the findings of previous studies, a destination’s image and tourists’ knowledge of it significantly affect their perceived value. This study tends to fill a gap in the literature and investigate this impact in crises-affected regions. Although there is a lack of research in this area, it is hypothesized that in unstable destinations, negative media portrayals can lower expectations, but first-hand knowledge or positive word-of-mouth can mitigate concerns, increasing perceived value. Moreover, novelty-seeking tourists and those motivated by adventure, exploration, and unique cultural experiences may perceive higher value in crisis-affected destinations due to the opportunity to visit less crowded, less commercialized sites. Thus, the following hypothesis is proposed:

*H*_*1*_ *: Knowledge and novelty have a positive impact on tourists’ perceived value in crisis-affected destinations.*

#### Curiosity and its impact on perceived value in tourism.

Curiosity is the desire to attain new knowledge and sensory experience that motivates exploratory behavior [90]. Many studies in the field of tourism tackled the main motives for travel, but a few tackled the concept of curiosity. Dunn Ross and Iso-Ahola [91] discussed curiosity as a motive to travel and similarly, Scott [92] found that curiosity favors first-time and repeat visitors. Also, Cha et al. [86] found that curiosity is one of the motives of Japanese tourists to see new places and learn new things. In the context of events and festivals, Kim and Lee [93] analyzed incentives for attending festivals and found that one of them is curiosity. In the context of tourism, Xu et al. [94] explored the motivation of tourists and found that curiosity is the basic motive. In their study, curiosity was found to be the first motive that influences what tourists could do in the destination. In other contexts, curiosity was found to have an effect on sports consumer behavior [95] and on purchase motivation [96]. Ciasullo et al. [97] who investigated curiosity in the tourism field and its effect on consumer behavior and loyalty stressed that curiosity plays a role in influencing tourist loyalty. The authors recommended that the role of curiosity should be magnified and reflected in marketing policies. Further, curiosity plays a significant role in travel-based learning, as it encourages tourists to actively engage with their surroundings and deepen their understanding of local cultures and histories [98]. Curiosity plays a critical role in motivating travelers to explore less conventional destinations [98], which can include those experiencing crises. The push-pull theory suggests that curiosity is a strong push factor driving individuals to overlook perceived risks in favor of authentic, immersive experiences [74]. Thus, although several studies tackled the concept of curiosity, only a few studies investigated the relationship between curiosity and perceived value in the context of tourism; and specifically none in the context of turbulent environments. Based on this gap in the tourism literature, the second hypothesis of this study is as follows:

*H*_*2*_ *: Curiosity has a positive impact on tourists’ perceived value in crisis-affected destinations.*

#### Attributes and their impact on perceived value in tourism.

In the context of tourism, attributes refer to the features and qualities of a certain destination [99,100]. Those features include natural beauty, historical significance, cultural heritage, leisure activities, and accessibility and transportation convenience. Many authors discussed the destination attractiveness and attributes and their impact on the tourism experience [101], quality of service experience and loyalty [102], and tourist satisfaction [103]. In addition, many authors such as Chi et al. [104], Murphy et al. [105], Pandža Bajs [106], Szubert et al. [107], Wu & Li [108], Yin et al. [109] and Zhang et al. [8] found that there is a positive relationship between destination attributes and perceived value. Further, Kim et al. [110] found that tourists’ perceived value is strongly influenced by the attributes of the destination, particularly those related to wellness, relaxation, and infrastructure. The authors highlighted that well-designed and appealing destination attributes enhance perceived value by providing fulfilling and high-quality experiences.

Beyond cultural and recreational attributes, environmental sustainability is increasingly shaping tourists’ perceived value and destination choices [111,112,113]. Green tourism and eco-friendly initiatives, such as sustainable accommodations and preserved natural landscapes are becoming key components of destination appeal [110,114]. Similarly, crisis-affected destinations can leverage their unique natural and cultural assets while integrating sustainability efforts to attract environmentally conscious tourists.

Middle Eastern countries are characterized by several attributes which contribute to the perceived value of tourists. They have diverse cultural heritages and historical sites which attract tourists. Also, they have their natural beauty and religious sites which are significant reasons for attracting tourists. Furthermore, some of these Middle Eastern countries have nightlife which plays a vital role in shaping the perceived value of tourists [115]. Thus, those attributes make the tourism experience in those countries special providing a multifaceted travel experience that distinguishes them from other places. Understanding those attributes and their influence on perceived value helps in formulating strategies to enhance Middle Eastern countries’ appeals to tourists.

In crisis-affected destinations, strong tourism infrastructure, secured areas, and protected cultural landmarks can reassure visitors and enhance their perception of value despite instability [14]. Some travelers may even perceive greater value in destinations that maintain cultural authenticity and historical significance despite economic or political struggles and this is the objective behind investigating the relationship between attributes and perceived value in the context of countries facing crises. Thus, the third hypothesis is as follows:

*H*_*3*_ *: Destination attributes have a positive impact on tourists’ perceived value in crisis-affected destinations.*

#### Value for money and its impact on perceived value in tourism.

In the service industry, value proposition delivery is the most significant factor for consumers. Value for money refers to a consumer’s perception of the utility of the product or service versus its opportunity and acquisition cost [116]. The five-dimension structure of value initiated by Petrick [66] includes monetary price. Thus, value for money is significant in perceived value and influences customer satisfaction and purchase intention. There are many strategies that companies consider to enhance customer value and specifically value for money. Customization is one of those strategies where companies offer tailored packages and personalized experiences [117]. Customization can upsurge perceived value by meeting specific tourist needs and preferences [118]. Moreover, creating bundled packages that include accommodation, meals, and activities can provide a sense of added value to tourists [119].

Many researchers discussed the role of the value of money in shaping the perceived value of tourists. In the hospitality sector, Tam [120] found that perceived costs have a direct negative effect on perceived value. In the context of wine tourism, Gill et al. [121] found that price has an impact on perceived value. Many authors such as Kashyap and Bojanic [122] and Gallarza and Gil Saura [67] stressed that perceived costs are the basis of perceived sacrifices and impact perceived tourist value.

Value for money is a critical determinant of perceived value in tourism [123]. By understanding and strategically enhancing value for money, tourism providers can enhance the perceived value of their offerings, thereby attracting and retaining more tourists. This involves not only competitive pricing but also maintaining high standards of quality and delivering memorable experiences that meet or exceed tourist expectations.

Economic crises often lead to currency devaluation and lower travel costs, making some crisis-affected destinations more affordable than stable alternatives [30]. The perceived value of a trip increases when tourists feel they are getting luxury or high-quality experiences at lower costs [67]. The EDT suggests that if travelers expect low-quality services but receive high-value experiences at a reduced cost, their satisfaction and loyalty increase [73]. Thus, based on the significance of the value of money in perceived tourist value, previous studies’ findings, and the objective of investigating the relationship between perceived value and value of money in turbulent environments, the fourth hypothesis is as follows:

*H*_*4*_ *: Value for money has a positive impact on tourists’ perceived value in crisis-affected destinations.*

#### Fun and family and their impact on perceived value in tourism.

Recreation and family are significant constructs for tourists and affect how they experience and perceive the world. In the context of tourism writing, ‘fun’ refers to the pleasure or happiness that people derive from recreational activities. Meanwhile, family tourism is defined as traveling with family members. Some research has focused on the impact of enjoying and engaging with family on various aspects of tourism. For instance, Tomić et al. [124] and Mihajlović and Koncul [125] found family holidays influence tourists’ satisfaction and their choice of destinations. They also discussed that family-friendly events are valuable for creating memories. These studies argue that fun and things that can be done with family are some of the key considerations that tourists have and their overall satisfaction with their tours.

Activities and facilities that are fun and suitable for families are increasingly becoming key factors defining the value of assets to tourists. Pomfret [126] added that whenever there are fun things for kids to do, it significantly enhances the perceived value of location for families. Siwek et al. [127] also discovered that when rooms and services are family-friendly, tourists are convinced that the place is worth more. The total utility of a tourism offering can increase if it has fun things to do and experiences aligned with the desires and preferences of various family members. It means that tourist spots and companies that engage in fun and family-oriented services may be more effective in enhancing tourists’ perceptions of value.

It is becoming clearer that fun and family are important in tourism, but there is still a lack of research on how they affect perceived worth, especially in the context of Middle Eastern countries’ tourism. Although these ideas have been looked at separately in studies, not many have directly examined how fun and family activities affect tourists’ views of Middle Eastern countries’ values. The attractions and cultural history of those Middle Eastern countries are very varied, so learning how fun and family affects how much something is worth could help the country’s tourism business.

Furthermore, fun and family-friendly experiences may become more attractive in crisis-affected destinations when enhanced security measures ensure safe zones for recreation. Moreover, some families perceive crisis-affected destinations as an opportunity to experience affordable vacations in places that would otherwise be more expensive. Based on what has already been written and the research gap that has been found, the following hypothesis was developed:

*H*_*5*_ *: Fun and family experiences have a positive impact on tourists’ perceived value in crisis-affected destinations.*

#### Escape and relaxation and their impact on perceived value in tourism.

As the principal purposes of travel, escape and recreation are the main concepts in tourism literature. Escape is to get away from the stress, while relaxation is looking for something different, new, and stress-free. Several papers have investigated these concepts within the context of tourism. For instance, Dean and Suhartanto [128] established that escape was one of the key motives for travel as it would compel the individual to attempt other forms of activities outside their usual environment. Similarly, Smith [129] also mentioned relaxation as one of the roles of tourism since it also allows guests to rejuvenate. These papers demonstrate how crucial escapism and leisure are in choosing the destination and how to spend the time.

Escape and relaxation, and perceived worth in tourism are intricately connected and have many-sided intersections. Offering tourists novelty and freedom through escape increases the product’s value in their total travel experience. The ability to escape the mundane nature of life offered by tourism made the tourists appreciate things much more, according to Abreu et al. [130]. While making the tourists feel good, relaxation, on the other hand, enhances how a place is perceived. According to Gadzali [131], pleasurable events enhance the satisfaction level of tourists and their perception of time. Whether a tourist location or product can meet these needs for escape and relaxation can make tourists feel that the location or the product is valuable.

Many research studies have talked about escape and rest and the satisfaction and experience of tourists. Still, not many have directly linked these ideas to perceived value, especially concerning Middle Eastern countries’ tourism. With their varied scenery and broad cultural offerings, Middle Eastern countries could be a place to get away and relax. Unfortunately, not enough research has been done on how these things affect tourists’ opinions of these destinations’ values. We can learn more about this topic by filling in the gaps in the existing research.

Tourists seeking mental and emotional relief from stress or routine life may find value in escaping to crisis-affected destinations where luxury tourism is affordable and less commercialized. The Seeking and Escaping Motivational Theory [76] explains that some tourists travel to escape personal difficulties and find therapeutic relaxation in destinations that offer wellness retreats, quiet nature, or cultural immersion. Based on the research that has already been done and the need for more detailed studies, this study comes up with the following hypothesis:

*H*_*6*_ *: Escape and relaxation have a positive impact on tourists’ perceived value in crisis-affected destinations.*

### The impact of perceived value on tourist satisfaction

The effect of perceived value on customer satisfaction was tackled by several authors. Many such as Abbasi et al. [132], Amri et al. [133], Chen [134], Chen and Chen [60], Chen and Tsai [135], Deng et al. [136], Eid [137], El-Adly and Eid [138], Gallarza and Saura [67], Hur et al. [139], Lee et al. [62], Petrick et al. [140], and Tu and Chih [141], found that perceived value positively influences visitors’ satisfaction, which in turn affects their loyalty. They found that tourists’ pleasure is significantly impacted by each of the fundamental aspects of their perceived value. This impacts the referrals of the services to others. In crisis-affected destinations, if tourists perceive higher value than expected whether through affordability, cultural and religious-sites richness, or safety measures, their satisfaction increases [30]. EDT suggests that if expectations are exceeded, tourists form positive impressions and are more likely to return [73]. Thus, based on the findings of these studies and the EDT, the following hypothesis was developed to be investigated in the context of crisis-affected regions:

*H*_*7*_ *: Satisfaction is positively influenced by perceived value in crisis-affected destinations.*

### The impact of satisfaction on tourist loyalty

A high level of satisfaction is generally associated with positive outcomes for destinations, including repeat visitation, positive word-of-mouth, and increased destination loyalty [142]. Hur et al. [139] and Tu and Chih [141] found that satisfied customers become loyal to a business and become less price-sensitive. Research in various settings, including tourism [132,137,138,67,62], hotels [143,61], and service organizations in general [144,145], found that satisfaction has a positive influence on loyalty. Satisfied tourists might want to revisit a destination and recommend it to others, even in politically or economically unstable conditions. Research on crisis-resistant tourism suggests that visitors who develop a positive emotional connection with a destination despite instability may demonstrate high loyalty [30]. Thus, based on the findings of these studies and the wide gap in the literature in the context of crises-prone regions, the following hypothesis is developed:

*H*_*8*_ *: Loyalty is positively influenced by satisfaction in crisis-affected destinations.*

## Research model

In the realm of consumer behavior and marketing, understanding the factors that influence perceived value is crucial for businesses aiming to optimize their offerings and enhance customer satisfaction and loyalty. Further, understanding the factors that shape perceived value is fundamental in tourism research, particularly in crisis-affected destinations where economic and political instability influence tourist decision-making.

**Fig 1:**
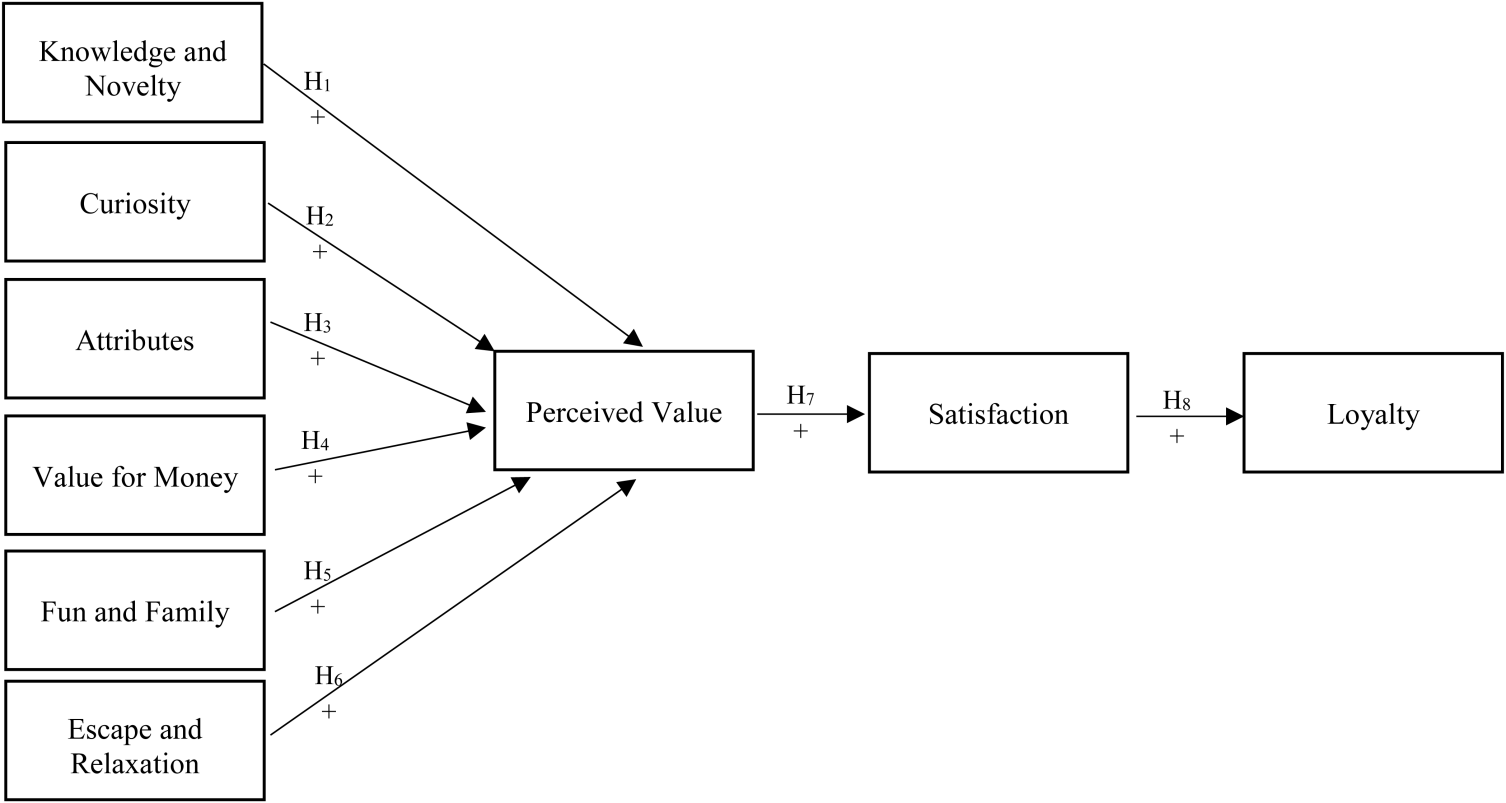
Research model.

Perceived value, a consumer’s overall assessment of the utility of a product based on what is received and what is given, is a multifaceted construct influenced by various elements. In the context of tourism and specifically in Middle Eastern countries facing crises, this study hypothesizes that multiple dimensions shape tourists’ perceived value, each contributing uniquely to a customer’s perception and ultimate satisfaction. Firstly, it is posited that knowledge and novelty (H1), as well as curiosity (H2), play significant roles in shaping perceived value. Attributes of the product (H3) and value for money (H4) are also expected to be key determinants. Additionally, experiential aspects such as fun and family (H5), and escape and relaxation (H6) are hypothesized to impact perceived value. Beyond these direct influences, the study further examines the downstream effects, suggesting that perceived value directly influences satisfaction (H7), which in turn affects customer loyalty (H8). These hypotheses collectively aim to provide a comprehensive understanding of the drivers of perceived value and their implications for customer satisfaction and loyalty in the context of tourism. The relationships between the variables are shown in Fig 1.

The research model is grounded in four key theoretical frameworks which are the Perceived Value Theory, EDT, Push-Pull Theory, and Iso-Ahola’s Seeking and Escaping Motivational Theory. These theories collectively explain how perceived value is formed, how it influences satisfaction, and ultimately, how satisfaction affects tourist loyalty. The Perceived Value Theory supports the hypotheses related to factors influencing perceived value (H_1_ to H_6_), such as knowledge and novelty, curiosity, fun and family, and value for money. Further, the Push-Pull Theory helps explain why certain factors drive tourists’ perceived value (H_1_ to H_6_). Knowledge and novelty (H_1_) and curiosity (H_2_) are push factors that motivate exploration and new experiences. Destination attributes (H_3_) and value for money (H_4_) act as pull factors that increase perceived value. Fun and family (H_5_) and escape and relaxation (H_6_) also align with push factors, influencing how tourists evaluate a destination’s worth in crisis conditions. Tourists visiting Lebanon may initially expect lower quality due to economic instability, but if they receive high-value experiences, affordability, or cultural enrichment, they may experience positive disconfirmation, leading to higher satisfaction. EDT supports the hypotheses that perceived value influences tourist satisfaction and loyalty (H_7_ and H_8_) emphasizing that satisfaction depends on whether a destination meets or exceeds expectations. In crisis destinations, tourists may lower their initial expectations, and if they receive a better-than-expected experience, their satisfaction and loyalty increase. Finally, Iso-Ahola’s Seeking and Escaping Motivational Theory provides a psychological explanation for why tourists visit crisis-affected destinations despite risks. This theory reinforces the role of push factors such as seeking religious, cultural, and wellness experiences and explains how some tourists willingly accept risks when the benefits of travel outweigh concerns about instability.

## Methodology

Our research strategy involved administering a structured questionnaire to tourists visiting countries facing crises, with data collection conducted face-to-face at various tourist sites. The questionnaire was made available in three languages—English, French, and Spanish—to cater to the predominant languages spoken by the diverse pool of international visitors. We ensured the accuracy and reliability of responses by involving a professional translator proficient in all three languages. The translator was not only qualified but was also thoroughly briefed on the research objectives to maintain consistency in interpretation. This step was crucial in eliminating language barriers and ensuring that the questions were understood uniformly across different respondents.

The questionnaire was carefully designed to capture the necessary data for the study. It consisted of two main sections: the first focused on demographic information, while the second targeted the measurement of the research variables using the Likert agreement scale (1 Strongly Agree to 5 Strongly Disagree). The constructs were measured using established scales from the literature and adapted to fit the context of the study. First, “knowledge-seeking and novelty” were measured using four statements adopted from the studies of Lee and Crompton [75] and Mangwane et al. [146]. Second, “value for money” was measured using four statements adopted from the studies of Caber et al. [5] and Mangwane et al. [146]. Third, “fun and family” were measured using four statements adopted from Caber et al. [5]. Fourth, “escape and relaxation” were measured using two statements adopted from Caber et al. [5]. Fifth, “attributes” were measured using five statements adopted from Pereira and Gosling [147]. Sixth, “curiosity” was measured using two statements adopted from the study of Martenson [148]. Seventh, perceived value was measured using five statements adopted from the study of Sweeney and Soutar [149]. Eighth, satisfaction was measured using four statements adopted from the study of Oliver [73]. Finally, loyalty was measured using four statements adopted from the study of Oliver [150]. These scales were chosen for their proven reliability and validity in previous studies. Before the full-scale data collection, we conducted a pilot study with 26 respondents to test the questionnaire’s effectiveness. Feedback from this pilot study led to minor adjustments in the wording of questions to improve clarity and ensure that they were culturally appropriate for the international tourist population.

We employed convenience sampling to gather cross-sectional data from April 2024 to June 2024. The collaboration with prominent travel agencies in Lebanon facilitated access to a wide range of foreign tourists, contributing to the diversity of our sample. Although convenience sampling has limitations, such as potential bias, our strategic partnership with the travel agencies helped mitigate these issues by targeting tourists at various points of their visit. Data was collected at multiple locations, including airports, hotels, and popular tourist attractions. Surveys with missing data were eliminated and the final sample size was 784 valid responses.

Lebanon was selected as the context for this research due to its unique and complex socio-political landscape, which has historically been marked by a series of crises and challenges [151]. The country has experienced prolonged periods of conflict, economic instability, and political turmoil, all of which have significantly impacted its tourism sector. Despite these adversities, Lebanon remains a culturally rich and geographically diverse destination with a strong appeal to both domestic and international tourists. This juxtaposition of ongoing challenges and persistent tourism activities makes Lebanon an ideal case study for understanding how destinations navigate and adapt to crises. This study used Covariance-Based Structural Equation Modeling (CBSEM) to test the relational hypotheses developed in the phase of data analysis. This process used an accurate dataset refinement process that excluded incomplete survey responses while determining outliers through z-standardized scores greater than three. Reliability was calculated through Cronbach’s alpha in addition to Composite Reliability (greater than 0.8) while determining validity through Average Variance Extracted (greater than 0.5) and Heterotrait-Monotrait ratio methods. Common Method Bias was determined by marking the variable technique. On analyzing raw scores, Perceived Value (4.28/5.0), Satisfaction (4.35/5.0), and Loyalty (4.12/5.0) indicated high mean scores, representing strong user positives in an era beset by crises in Lebanon.

Regarding the ethical considerations, we went beyond maintaining anonymity and confidentiality. Each respondent was provided with a detailed information sheet explaining the purpose of the study, how their data would be used, and their rights as participants. They were assured that participation was entirely voluntary and that they could withdraw at any time without any consequences. Additionally, data protection measures were rigorously applied, including secure storage of the completed questionnaires and the use of encryption software for digital data handling. Further, we obtained ethical approval from the Research Ethics Committee at the Holy Spirit University of Kaslik (USEK) before data collection to maintain ethical considerations.

Table 1 shows the sample profile.

**Table 1:** Sample Profile.

Demographic Variable	France (241)	Spain (86)	Italy (55)	Portugal (43)	U.S.A (55)	U.K. (54)	Germany (31)	Australia (70)	Japan (24)	China (49)	Others (77)
**Gender**											
Male	58.09%	41.86%	47.27%	60.46%	48%	44.45%	49%	50%	47%	46.93%	41.02%
Female	41.90%	58.13%	52.72%	39.53%	52%	55.55%	51%	50%	53%	53.06%	58.98%
**Age Group**											
18-25	7.4%	8.13%	7.27%	8.15%	9.1%	7.40%	8%	9%	7%	0%	4.57%
26-35	18.25%	20.93%	21.81%	8.15%	22.5%	5.85%	19%	20%	21%	0%	21.48%
36-45	22.40%	23.25%	32.72%	30.23%	30%	0%	29%	28%	27%	26.53%	16.15%
46-55	21.99%	30.23%	16.36%	16.27%	18.4%	26.43$	23%	22%	24%	32.66%	17.57%
56+	29.87%	19.09%	21.81%	37.20%	20%	57.40%	21%	21%	21%	40.81%	40.23%
**First Time Visiting the Country**	92.53%	100%	94.54%	93.02%	95%	90.74%	94%	96%	95%	100%	96.48%
**Second Time Visiting the Country**	74.68%	0%	5.46%	6.98%	5%	9.25%	6%	4%	5%	10%	3.51%

## Results

### Measurement model

Before analysis, the collected data underwent a comprehensive cleaning process to ensure accuracy and reliability. Surveys with missing responses for key variables and incomplete demographic information were excluded from the final dataset. Outliers were identified using standardized z-scores, with values beyond ±3 standard deviations examined and removed if they resulted from data entry errors or skewed results. Logical consistency checks were performed to ensure that responses aligned with expected patterns, with any inconsistencies clarified or excluded if unresolved.

The study employed several statistical techniques to analyze the data. Descriptive statistics, including means, standard deviations, and frequency distributions, were calculated to summarize the demographic profile and key variables. Confirmatory Factor Analysis (CFA) was conducted using Covariance-Based Structural Equation Modeling (CBSEM) to assess the reliability and validity of measurement scales, with fit indices including the Chi-square/df ratio, RMSEA, CFI, and SRMR. Internal consistency reliability was evaluated using Cronbach’s alpha and Composite Reliability (CR), while convergent validity was assessed using the Average Variance Extracted (AVE) to confirm that each construct captured sufficient variance from its indicators. Discriminant validity was verified using the Heterotrait–Monotrait Ratio (HTMT) to ensure distinctiveness between constructs. To test for Common Method Bias (CMB), the marker variable method was employed, using social media usage as the marker variable. Structural Equation Modeling (SEM) was applied to test the hypothesized relationships between latent variables, with standardized coefficients and p-values reported.

A snapshot of key raw data points illustrates the distribution of responses across core constructs. For example, the mean score for Knowledge and Novelty was 3.91 (SD = 0.82) on a scale of 1–5, while Curiosity had a mean of 4.02 (SD = 0.78). Destination Attributes averaged 4.25 (SD = 0.71), and Value for Money scored 4.34 (SD = 0.66). Fun and Family experiences had a mean score of 4.17 (SD = 0.75), and Escape and Relaxation averaged 4.22 (SD = 0.68). Perceived Value was rated at 4.28 (SD = 0.72), Satisfaction at 4.35 (SD = 0.69), and Loyalty at 4.12 (SD = 0.81). These scores reflect high levels of perceived value, satisfaction, and loyalty, suggesting that tourists had positive experiences despite the destination’s crisis context.

Outlier detection and treatment were specifically conducted using standardized z-scores, with values beyond ±3 standard deviations removed if they resulted from data entry errors or significantly skewed the results. This approach ensured that the dataset remained representative while excluding anomalies that could bias the analysis.

The first phase of the analysis involved an examination of the measurement model’s characteristics, focusing on the reliability and validity of the employed measures. Covariance-based structural modeling (CBSEM) was selected as the analytical method to scrutinize the intricacies of the measurement model.

A confirmatory factor analysis (CFA) was conducted to assess the fit of the measurement model and validate the reliability and validity of the measures. The results revealed that the measurement model exhibited an acceptable fit to the data, taking into consideration the sample size (χ2/df = 1.74; p = .00), RMSEA = 0.06 [CI:.06 −.07], CFI = 0.9; SRMR = 0.06).

The measures in Table 2 demonstrated commendable levels of internal consistent reliability, as evidenced by Cronbach’s alpha (> 0.7) and composite reliability (> 0.8). Furthermore, the indicators exhibited significant loadings (p < 0.01) on their respective measures, with no discernible cross-loadings, thereby affirming robust support for the convergent validity of the measures.

**Table 2:** Reliability and validity.

	CR	AVE
Knowledge and Novelty	0.91	0.64
Curiosity	0.92	0.61
Attributes	0.94	0.78
Value for Money	0.95	0.66
Fun and Family	0.91	0.72
Escape and Relaxation	0.90	0.57
Perceived Value	0.93	0.74
Satisfaction	0.92	0.65
Loyalty	0.93	0.62

The average variance extracted (AVE) of the measures in Table 2 surpassed the predetermined cutoff level of 0.5, underscoring the satisfactory discriminant validity of the measures.

Utilizing the heterotrait–monotrait ratio (HTMT) method, we examined the measures’ discriminant validity. All inter-factor HTMT values fell below the 0.85 threshold, providing strong evidence in favor of the discriminant validity of the measures. To preclude the possibility of inflating or deflating variance due to common method bias (CMB), procedural remedies were deployed, such as the separation of predictor and criterion constructs and the use of different scales, as elucidated in the seminal work of Podsakoff et al. [152].

To ascertain the potential presence of CMB, the marker variable method [153] was employed, with participants’ use of social media as the designated marker variable. The measure exhibited no significant correlation with other variables in the model. The inclusion of the marker variable did not yield significant changes in the results of the hypothesized model, providing compelling evidence of the absence of common method bias in this study.

### Structural model

The suitability of the dataset for structural equation modeling was confirmed by checking for multivariate assumptions. To ensure inter-constructs have interrelationships in accordance with theoretical expectations, the inter-construct intercorrelation matrix establishes discriminant validity according to HTMT standards by representing inter-construct intercorrelation by similarities and dissimilarities between constructs.

The structural model tested encompassed all direct effects among latent variables in the hypothesized model. The analysis revealed that the structural model exhibited an acceptable fit to the data (χ2/df = 2.2; p = .00), RMSEA = 0.07 [CI95%:.07 –.08], CFI = 0.9; SRMR = 0.06). Standardized coefficients for all paths were significant at p < 0.01, except for the paths from knowledge and novelty and curiosity to perceived value, which were not significant. Overall, these validation steps ensure the reliability, validity, and robustness of the results.

The correlation matrix (Table 3) illustrates the relationships between key constructs in the study. All correlations were significant at p < 0.05, except for the relationships between loyalty and other constructs, which were weak and non-significant. Notably, perceived value exhibited moderate-to-strong correlations with attributes (r = 0.44), value for money (r = 0.52), fun and family (r = 0.44), and escape and relaxation (r = 0.59), supporting the importance of these factors in shaping perceived value. The positive correlation between perceived value and satisfaction (r = 0.55) aligns with previous research, reinforcing the notion that higher perceived value leads to greater satisfaction. Additionally, satisfaction demonstrated a moderate correlation with loyalty (r = 0.46), highlighting its role in fostering repeat visits and recommendations. Thus, the correlation matrix underscores the study’s findings that attributes, value for money, fun and family, and escape and relaxation are key determinants of perceived value, while knowledge, novelty, and curiosity play a lesser role in the context of crisis-affected destinations.

**Table 3:** Correlation matrix.

	KN	CU	ATT	VM	FF	ER	PV	SAT	LO
Knowledge and Novelty (KN)	.69								
Curiosity (CU)	.44^*^	.67							
Attributes (ATT)	.42^*^	.55^*^	.86						
Value for Money (VM)	.38^*^	.5^*^	.46^*^	.84					
Fun and Family (FF)	.61^*^	.36^*^	.28^*^	.29^*^	.92				
Escape and Relaxation (ER)	.32^*^	.45^*^	.44^*^	.46^*^	.34^*^	.88			
Perceived Value (PV)	.28^*^	.49^*^	.44^*^	.52^*^	.44^*^	.59^*^	.74		
Satisfaction (SAT)	.24^*^	.47^*^	.39^*^	.46^*^	.35^*^	.54^*^	.55^*^	0.72	
Loyalty (LO)	−.04	−.12	−.14^*^	−.07	−.05	−.2^*^	.03	−.08	1

Significance level:

*p < 0.05.

The results from the structural model in Table 4 indicated several relationships among latent variables. Positive relationships were observed between attributes (β = .4; p < .01), value for money (β = .42; p < .01), fun and family (β = .34; p < .01), escape and relaxation (β = .38; p < .01), and tourist perceived value (β = .46; p < .01). These findings provided support for hypotheses H_3_, H_4_, H_5_, and H_6_. However, knowledge and novelty (β = .26; p = 0.4) and curiosity (β = .55; p = 0.72) did not exhibit significant impacts on tourist perceived value. Consequently, hypotheses H_1_ and H_2_ were rejected based on these results. Furthermore, a positive relationship was identified between perceived value and satisfaction (β = .22; p < .01), confirming support for hypothesis H_7_. The structural model results also revealed a significant positive relationship between satisfaction and loyalty (β = .61; p < .01), supporting hypothesis H_8_.

**Table 4:** Structural model results.

Direct Effect	Standardized Coefficient
KN → PV	.26
CU → PV	.55
ATT → PV	.4^**^
VM → PV	.42^**^
FF → PV	.34^**^
PV → SAT	.38^**^
SAT → LO	0.46^**^

Note:

**p < .01.

## Discussion

The findings of this study provide valuable insights into the determinants of tourists’ perceived value and its subsequent impact on satisfaction and loyalty, particularly within the context of destinations facing challenges and crises. In such environments, where political instability, economic difficulties, or social unrest might deter tourism, understanding how to enhance perceived value is crucial for maintaining and even growing the tourism sector.

The significant positive impact of destination attributes, such as natural beauty, cultural heritage, and accessibility, on tourists’ perceived value underscores the universal importance of these features. This is consistent with prior research [104,105,8], which has highlighted that these inherent qualities are central to tourists’ evaluations of any destination. However, in destinations facing crises, these attributes take on an even greater significance. For example, promoting the unique cultural heritage of a crisis-affected destination can serve as a powerful draw for tourists seeking authentic and meaningful experiences, offering a sense of continuity and resilience that contrasts with the surrounding challenges. Additionally, enhancing accessibility through improved infrastructure or targeted marketing campaigns that reassure tourists of safe and reliable travel can help mitigate the negative perceptions associated with a crisis-impacted area.

Value for money emerged as a critical determinant of perceived value, aligning with the findings of Gallarza and Gil Saura [67], Gill et al. [121], and Tam [120]. In destinations facing economic or political challenges, tourists are often more price-sensitive and may be looking for greater assurances that their investment in travel will be worthwhile. Thus, competitive pricing strategies that do not compromise on quality become essential. In crisis-affected destinations, where the local economy might be struggling, offering high-quality experiences at a reasonable cost can not only attract tourists but also contribute to the economic recovery of the area. The ability to deliver value for money in such contexts can significantly enhance perceived value, encouraging repeat visits and positive word-of-mouth, which are crucial for sustaining tourism in difficult times.

The role of fun and family experiences in enhancing perceived value further emphasizes the importance of catering to tourists’ desire for enjoyable and memorable experiences. This aligns with the research of Mihajlović and Koncul [125], Pomfret [126], Siwek et al. [127], and Tomić et al. [124]. In destinations facing challenges, providing family-oriented and recreational activities can create a sense of normalcy and happiness that contrasts with the crisis environment, making the destination more appealing. This approach not only enhances perceived value but also helps in repositioning the destination as one that is resilient and capable of offering positive experiences despite its challenges. For instance, creating safe, family-friendly zones or organizing cultural festivals can serve as a counter-narrative to the crisis, attracting families and groups who might otherwise be hesitant to visit.

Escape and relaxation were also found to be principal drivers of perceived value, echoing the findings of Abreu et al. [130], Dean and Suhartanto [128], Gadzali [131], and Smith [129]. In the context of crisis-affected destinations, the desire for escape and relaxation can be particularly pronounced. Tourists visiting these areas may be looking for a refuge from their own stresses or seeking the unique tranquility that such destinations can offer. Leveraging the natural landscapes, tranquil settings, and cultural serenity that are often found in these regions can be a strategic way to enhance perceived value. Marketing campaigns that emphasize the peacefulness and rejuvenating qualities of the destination can resonate with potential visitors, particularly those looking for a break from their routine or the stresses of life in more stable regions.

Contrary to expectations, knowledge and novelty did not significantly impact perceived value in this study. This divergence from prior studies [84,85] suggests that in crisis-affected destinations, tourists may prioritize emotional and experiential factors over intellectual engagement or newness. While knowledge and novelty can be significant draws in more stable contexts, they may be less critical in destinations where safety, comfort, and enjoyment are more immediate concerns. This finding suggests that tourism marketing in crisis-affected destinations should focus more on the emotional and experiential aspects of the visit—such as relaxation, enjoyment, and family bonding—rather than solely promoting novel experiences or educational opportunities.

Similarly, curiosity did not show a significant impact on perceived value, which contrasts with the theoretical assertions of Silvia [90] and Ciasullo et al. [97]. This outcome suggests that while curiosity might drive initial interest in a crisis-affected destination, it does not necessarily translate into a higher perceived value once the destination is experienced. For tourists, the reality of the experience—such as the quality of service, safety, and overall enjoyment—may outweigh the initial curiosity that led them to consider the destination. Therefore, marketing efforts might benefit from focusing on delivering tangible, positive experiences rather than relying on the intrigue of the destination alone.

The study further confirms that perceived value positively influences satisfaction, which in turn enhances loyalty. These findings are consistent with the established literature [132,133,135,139]. In the context of crisis-affected destinations, where attracting and retaining tourists can be particularly challenging, this relationship underscores the importance of continuously enhancing perceived value. High perceived value leads to greater satisfaction, which encourages tourists to return and recommend the destination to others. For tourism sectors in such challenging environments, a strategic focus on attributes like value for money, family experiences, and relaxation can help build a loyal tourist base. This loyalty is not only crucial for sustaining tourism in the short term but also for aiding the long-term recovery and resilience of the destination.

## Theoretical implications

This study intended to investigate the different factors affecting the perceived value of tourists and the effects on satisfaction and loyalty in crisis-affected regions using four theories. The findings of the study not only validate those theories in the context of crisis-affected destinations but also extend their applicability by showing how certain factors interact to shape tourists’ perceived value, satisfaction, and loyalty.

First, the findings showed that destination attributes and value for money have a positive impact on perceived value which reinforces the applicability of the Perceived Value Theory in crisis-affected regions by highlighting that in such destinations, value is assessed through a more complex lens where factors such as affordability and attributes become more significant. Thus, it can be noted that tourists adjust their perceptions of value based on the affordability, uniqueness, and resilience of the tourism sector in crisis settings. This calls for an expanded conceptualization of perceived value that includes risk-adjusted perceived value, where tourists weigh affordability and cultural significance against potential safety concerns.

Second, the findings that satisfaction is influenced by perceived value and loyalty is influenced by satisfaction confirm the EDT which plays a vital role in such environments. The results suggest that in unstable environments, tourists tend to adjust their expectations downward due to negative perceptions shaped by economic or political instability. However, when their experiences exceed these initial expectations through affordability or cultural and religious richness tourist satisfaction and loyalty increase. The findings suggest that expectation management strategies such as accurate pre-visit information, transparency in communication, and destination branding can actively shape disconfirmation experiences, either mitigating dissatisfaction or amplifying positive surprise effects.

Third, the findings showed that fun and family and escape and relaxation have a positive impact on perceived value which aligns with the Push-Pull Theory highlighting the idea that tourists balance their internal motivations or push factors and destination attractiveness or pull factors even in crisis settings. This study extends the theory by showing that certain pull factors such as cultural heritage, religious sites, and affordability gain heightened importance in crisis destinations, as tourists prioritize cost-effective and meaningful experiences over traditional luxury offerings. Furthermore, the results suggest that push motivations can be amplified in unstable destinations. For instance, travelers seeking relaxation may view a crisis-affected destination as a low-cost alternative to traditionally expensive resorts, while those looking for adventure or novelty may be drawn to destinations that offer a unique or unconventional experience. This highlights the need for a nuanced understanding of risk-adjusted motivations, where tourists are not simply repelled by instability but may instead reframe risks as part of the adventure or cost-benefit equation.

Finally, the results show strong support for Iso-Ahola’s Seeking and Escaping Motivational Theory. Specifically, the results explain why tourists continue to visit crisis-affected destinations. The findings expand this theory by highlighting how tourists recalibrate risk perception based on their individual motivations. For instance, travelers seeking religious or cultural enrichment may prioritize these experiences over safety concerns, while those escaping economic constraints may see a crisis-affected destination as an opportunity for affordable luxury or budget-friendly travel. Also, the findings challenge the assumption that risk universally discourages travel and instead suggest that perceived risk is relative rather than absolute. Destinations facing crises can leverage this insight by targeting specific tourist segments who rationalize risk differently and may be drawn to the emotional, cultural, religious, or financial benefits that outweigh their concerns.

By integrating these four theories, this study provides a comprehensive framework for understanding tourism behavior in crisis contexts, bridging gaps in previous studies that primarily focused on stable tourism destinations. These insights not only refine the application of existing theories but also offer strategic directions for destination managers and policymakers seeking to enhance tourism resilience in challenging environments.

## Managerial implications

The research findings present significant contributions to knowledge of tourists’ perceived value in crisis destinations with significant implications for policymakers, destination managers, and stakeholders in the tourism industry. The research contributes to building knowledge by identifying determinants of perceived value in risky environments, a previously unexamined subject in tourism literature. By deviating from the usual drivers of perceived value like novelty and knowledge and firmly focusing on value for money, enjoyment and family experience, and escape and relaxation, the study contributes at an advanced level to conceptualization in the tourism resilience of crisis destinations.

The research concludes that value for money is high on the agenda for destination managers to ensure tourists visit and remain in crisis-affected destinations. Economic adversity usually means devaluation, which attracts foreign tourists to crisis-affected destinations due to the lower (convenient) prices. Despite the apparent preferences of the tourists, managers must ensure high-quality services. Investment in security devices, service reliability, and infrastructure guarantees the guests’ satisfaction and future commitment [154]. Further, managers should ensure excellence and customization of services to create and sustain value perception and positive brand equity for the hotel destinations [155]. These insights infer that managers in crisis-affected tourist destinations should implement strategies to enhance the value of money for the tourists.

The findings show that during crises, tourists’ preferences are driven by emotional and experiential factors and not conventional tourism drivers like novelty. The study emphasizes the demand for family-friendly leisure and recreation activities as the major drivers of tourism activities. It, therefore, logically follows that managers must design customized experiences, such as family recreational activities, safe zones for amusement, and engagement in cultural activities, to suit the tourists’ needs and preferences. According to Nyadzayo and Khajehzadeh [156], in customer relationship management, customer satisfaction leads to emotional engagement and, ultimately, customer loyalty [156]. The findings also show that searching for escape and relaxation are critical motivators of perceived value. The inference is that crisis-affected destinations should focus on well-being tourism, nature escapes, and cultural immersion. This aligns with post-pandemic changes in tourism, where tourists seek quiet destinations that bring mental relaxation and freedom. Marketing campaigns must, therefore, highlight the destination’s ability to offer peace in the face of extrinsic disturbance, reinforcing the psychological appeal of being there.

Crisis-affected tourist destinations are usually subjected to negative media attention, adversely affecting their brand image. The company must communicate the crisis to restart destination stories and market resilience, safety, and quality tourism experiences. Brand equity research emphasizes that open and ongoing communication can develop confidence and efficiently diminish perceived risk. Therefore, Destination marketing organizations (DMOs) must include emerging media like social networking, influencer marketing, and live customer feedback functions to enhance brand image and gain tourist credibility [154]. It is the best means to establish long-term destination loyalty and offset the negative effects of political or economic crises.

Multi-stakeholder collaboration is critical to sustaining tourism in destinations plagued by crises. Policymakers, tourism establishments, and stakeholders at the destination level need to collaborate in framing policies that maximize the value for tourists. Public Private Partnerships (PPPs) can enhance tourism infrastructure, safety, and sustainable demand for tourism. Moreover, service delivery through technology, such as AI interface and customized digital experience, can also enable value perception building and destination brand empowerment [155,156]. Sustained demand by tourists can be achieved by focusing on service quality, affective loyalty, stakeholder coordination, and strategic brand management.

## Limitations and further studies

This study provides valuable insights into tourists’ perceived value in crisis-affected regions; however, it is not without limitations. First, this study is limited to Lebanon as a single tourism context, restricting the generalizability of the results to other crisis-affected regions. Future studies could consider comparative studies across multiple crisis-affected regions to examine whether cultural, economic, and geopolitical differences alter the factors influencing tourist perceived value. Second, demographic differences were not incorporated in the model of this study. Thus, future studies can consider variables such as occupation, education level, income, and cultural background which might impact tourists’ perceptions and decision-making in turbulent destinations. Future research could also investigate whether different demographic groups perceive and evaluate crisis-affected tourism differently. Third, this study overlooks technological factors that shape tourists’ experiences. As digitalization transforms the tourism industry, future studies could examine the impact of E-scape, E-responsiveness, and virtual tourism innovations on perceived value, satisfaction, and loyalty in crisis-affected destinations. Fourth, this study does not explore brand equity, even though loyalty is an essential outcome variable in the model. Future research could extend the model to assess whether higher perceived value and tourist satisfaction contribute to stronger destination brand equity, particularly in unstable regions. Further, as a cross-sectional study, this research captures a snapshot of tourist perceptions at a single point in time. A longitudinal study could provide deeper insights into how tourists’ perceptions, satisfaction, and loyalty evolve before, during, and after a crisis. Additionally, while this study addresses perceived value in unstable environments, it does not account for environmental sustainability factors such as green tourism, carbon footprint concerns, and climate adaptation efforts. Future research could examine whether tourists visiting crisis-affected destinations also consider eco-tourism initiatives and sustainable travel practices in their decision-making. Finally, more studies are needed to find out whether similar findings apply to other non-stable countries by identifying travel motivation variations by type of destination, examining motivations by clusters of travelers, and conducting market segmentation of seekers and escapers. By addressing these limitations and expanding future research directions, researchers can build on this study to gain a more comprehensive understanding of tourist behavior in crisis-affected destinations.

## Supporting information

S1 File Copy of Final Responses. (XLSX)
